# Stress-Induced Changes in Autonomic Activity and Fascia Hydration: A Cross-Recurrence Quantification Analysis in a Naturalistic Exam Paradigm

**DOI:** 10.3390/jcm15207839

**Published:** 2026-10-11

**Authors:** Andreas Brandl, Lea Overmann, Robert Schleip, Glen Farrugia, Tobias Nowy, Christian Helmrich

**Affiliations:** 1 Conservative and Rehabilitative Orthopedics, TUM School of Medicine and Health, Technical University of Munich, 80333 Munich, Germany; robert.schleip@tum.de; 2 Department of Health and Social Sciences, Hochschule Fresenius, 20148 Hamburg, Germany; 3 Association for Fascia Research, 80799 Munich, Germany; dr.leaovermann@gmx.de; 4 Department for Medical Professions, Diploma Hochschule, 37242 Bad Sooden-Allendorf, Germany; 5 School of Health and Psychology, Triagon Academy, MRS 1331 Marsa, Malta; glen.farrugia@triagon.mt (G.F.); tobias.nowy@triagon.mt (T.N.); 6 Department of Psychology, SRH Fernhochschule, 88499 Riedlingen, Germany

**Keywords:** exam stress, thoracolumbar fascia, hydration, bioelectric impedance analysis, heart rate variability, cross-recurrence quantification analysis

## Abstract

**Background:** The autonomic nervous system is increasingly recognized as a key regulator of fascial physiology. Interventions that modulate autonomic activity, including breathing techniques, relaxation strategies, and movement-based therapies, have therefore been proposed to influence tissue hydration and mechanics, as well as functional outcomes, yet evidence under real-life stress conditions remains limited. This study examined the effects of acute exam stress on autonomic activity, lumbar tissue hydration and their dynamic interaction. **Methods:** Eighteen students (9 female, 9 male; 36.1 ± 11.6 years) were assessed in front of peers and an examiner as part of an osteopathic medical school training (exam condition) and again after a four-week recovery period (control condition). Heart rate variability (HRV) and local bioelectrical impedance analysis (BIA) of the lumbar region were recorded. Subjective stress over the past four weeks was measured using the Perceived Stress Scale (PSS-10). Interactions between HRV and BIA were analyzed using cross-recurrence quantification analysis (CRQA). **Results:** Compared to the control condition, the exam condition showed significantly increased CRQA values (0.38 ± 0.44 vs. −0.33 ± 1.27; *p* = 0.037), indicating stronger system coupling. HRV was significantly reduced (716 ± 141 ms vs. 813 ± 105 ms; *p* < 0.001), reflecting increased autonomic activation. BIA values were also significantly lower (24.55 ± 9.28 Ω vs. 30.25 ± 9.77 Ω; *p* = 0.015), suggesting altered tissue hydration. PSS-10 scores were significantly higher during the exam condition (22.63 ± 5.46). **Conclusions:** Acute psychosocial stress appears to induce measurable changes in autonomic regulation and lumbar tissue properties and enhances their dynamic interaction. These findings provide initial indication for the concept of autonomic-fascial coupling and emphasize the relevance of integrative, biopsychosocial approaches in clinical physical therapy.

## 1. Introduction

The field of clinical physical therapy is currently undergoing a profound transformation, driven by demographic changes, the increasing prevalence of chronic musculoskeletal disorders and the growing burden of persistent pain conditions [1]. These challenges increasingly demand a shift from purely biomechanical models toward integrative, biopsychosocial approaches that account for the complex interplay between physiological, psychological and behavioral processes [2]. Within this evolving framework, psychosocial stress has emerged as a central factor influencing both systemic regulation and tissue-level function [3,4].

Stress is widely understood as a nonspecific physiological response to internal and external demands, which is shaped by cognitive appraisal and perceived controllability [5,6]. Acute stress, such as that occurring during examination situations, activates key biological systems, particularly the autonomous nervous system (ANS) and the hypothalamic-pituitary-adrenal (HPA) axis. These responses lead to the release of catecholamines and cortisol and are associated with cardiovascular, metabolic and immunological changes [7,8]. While such responses are adaptive in the short term, repeated or excessive activation may contribute to systemic dysregulation, increased inflammatory activity and heightened risk of chronic disease [9,10].

Beyond systemic effects, growing evidence suggests that stress also directly influences peripheral tissues, including the myofascial system. The fascia represents a highly interconnected, mechanosensitive network that integrates structural, sensory and regulatory functions [11]. In particular, the thoracolumbar fascia (TLF) has gained increasing attention as a potential mediator between autonomic regulation and biomechanical function. This structure is densely innervated, strongly influenced by autonomic activity and critically dependent on the hydration state of its extracellular matrix, which determines its viscoelastic and shear properties [12,13].

Recent interdisciplinary research at the intersection of neuroscience, psychophysiology and mind–body medicine has further highlighted the bidirectional nature of regulation between the central nervous system and peripheral tissues. Top-down processes, such as cognitive appraisal and emotional responses, interact with bottom-up signals arising from somatic and interoceptive input, contributing to the regulation of physiological states and pain perception [4,14,15]. In this context, stress can be conceptualized not only as a psychological experience but also as a systemic regulatory process that probably modulates tissue function. This perspective is consistent with contemporary models of clinical physical therapy that emphasize integrative, patient-centered approaches to complex chronic conditions [16].

A key mechanism linking stress and tissue properties may involve stress-induced changes in microcirculation and extracellular matrix hydration. Activation of the ANS can alter vascular tone and capillary filtration, leading to rapid shifts in interstitial fluid distribution [17,18]. Because the mechanical properties of fascia are strongly influenced by fluid content and hyaluronan-mediated lubrication, such changes may directly affect tissue stiffness, sliding capacity and force transmission [13,19]. These processes are particularly relevant in the lumbar region, where the TLF plays a central role in load transfer, movement coordination and pain modulation [20,21].

Alterations in the biomechanical properties of the TLF have been associated with musculoskeletal disorders, especially nonspecific low back pain, which accounts for a substantial proportion of global disease burden [1,12]. Emerging evidence further suggests that stress-related changes in fascial stiffness, hydration and viscoelasticity may contribute to both the onset and maintenance of such conditions [14,22]. These findings support the hypothesis that the myofascial system is a key interface between psychological stress and physical health, reinforcing the importance of considering systemic regulation in clinical physical therapy.

Despite these advances, empirical evidence examining the acute effects of psychosocial stress on local tissue properties remains limited. In particular, the simultaneous assessment of autonomic regulation and tissue hydration—and their dynamic interaction—has rarely been investigated under ecologically valid conditions. Non-invasive methods such as heart rate variability (HRV), which can provide insight into autonomic activity [23], and local bioelectrical impedance analysis (BIA), which reflects local tissue hydration [24], offer promising tools to address this research gap. Furthermore, nonlinear analytical methods such as cross-recurrence quantification analysis (CRQA) enable investigation of complex dynamic interactions among physiological systems that go beyond traditional correlation-based approaches [25,26].

Against this background, the present study investigates the effects of acute exam stress as a real-life psychosocial stressor on autonomic regulation, lumbar tissue hydration and subjective stress perception. By integrating HRV, BIA and CRQA-based system coupling analysis, this work aims to explore the primary hypothesis that acute stress induces measurable, short-term changes in myofascial tissue dynamics and enhances the functional coupling between autonomic activity and peripheral tissue processes. A secondary hypothesis is that this coupling is dependent on the level of perceived subacute/chronic stress.

## 2. Materials and Methods

This study was conducted using an observational repeated-measures design in which participants were assessed under two conditions: an acute stress condition and a control condition.

Artificial intelligence (AI) tools were used solely for language editing and stylistic refinement of the manuscript text. The authors reviewed and verified all generated content and take full responsibility for the final version of the manuscript.

### 2.1. Setting and Participants

The sample size was calculated based on a previous study in which the CRQA methodology was tested using physiological data from the thoracolumbar myofascial system [27], assuming a dropout rate of 10% (d = 0.9, α err = 0.05, 1 − β err = 0.9) [28]. A total of 18 osteopathy students, comprising 9 female and 9 male participants, aged between 24 and 58 years, were included in the study. Participants were recruited from the third semester of a Bachelor’s degree program.

Eligibility criteria required participants to be between 18 and 60 years of age and to report a good or very good general health status, as defined by the WHO [29]. Exclusion criteria comprised chronic inflammatory diseases, cardiovascular disorders including hypertension, the use of medication affecting cardiac function, intake of muscle relaxants, dermatological conditions in the lumbar region, scars within the region extending from thoracic vertebra T12 to sacral vertebra S1, musculoskeletal disorders within the previous six months, and psychiatric or psychotic disorders within the past five years.

Participants were examined under two different conditions. The exam condition represented an acute stress situation and consisted of a real academic examination setting. Participants delivered a 15-min oral presentation in front of an audience of approximately 25 individuals, including peers and an examiner. This context was considered a naturalistic psychosocial stressor. Immediately prior to the presentation, participants completed the Perceived Stress Scale (PSS-10) [30]. Subsequently, electrodes for HRV and BIA measurements were applied. Data recording took place during the central phase of the presentation, specifically between minutes five and ten.

The control condition was conducted four weeks after the examination, following a recovery phase without examination-related stressors. Measurements were conducted in a low-stress teaching environment, with participants standing upright and engaging in informal conversation with peers. The measurement duration and procedures were identical to those used in the stress condition to ensure comparability.

### 2.2. Measured Outcomes

HRV was recorded using a validated chest strap sensor (Polar H10, Polar Electro, Kempele, Finland) with a sampling frequency of 1000 Hz [31]. The complete sequence of individual, beat-to-beat RR intervals were extracted using Kubios HRV software (Version 3.5.0) [32]. HRV was used as a non-invasive indicator of autonomic nervous system activity, reflecting changes in the autonomic nervous system under various psychosocial conditions [23].

**Figure 1:**
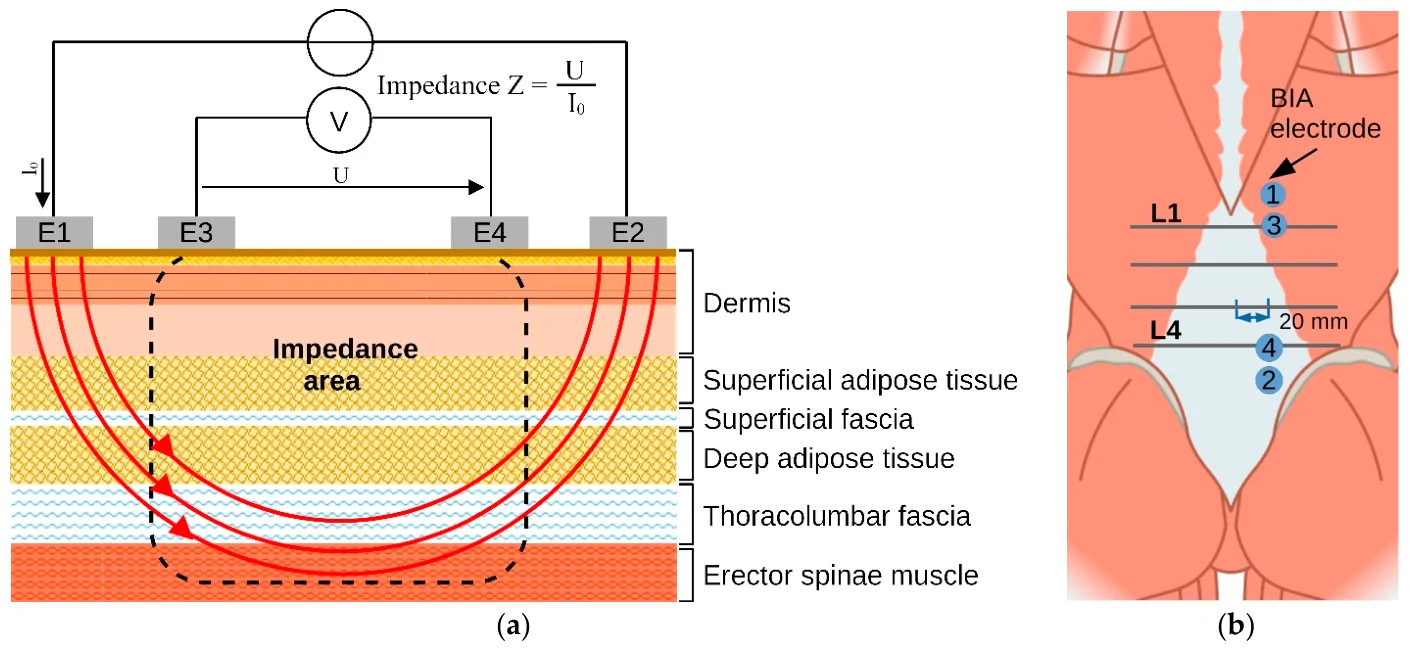
BIA Measurement Setup. (**a**) BIA measurement with four electrodes. E1, E2, current electrodes; E3, E4, voltage electrodes; V, voltmeter; U, voltage; I0, measurement current. (**b**) Measurement Setup. 1, 2, current electrodes; 3, 4, voltage electrodes.

BIA was assessed using a four-electrode system (Fascia-Impedance THU instrumentation, Ulm University of Applied Sciences, Ulm, Germany). A constant current of 20 µA at a frequency of 12 kHz was applied. This relatively low measurement frequency was selected because, at lower frequencies, cell membranes exhibit substantial capacitive reactance and therefore impede current flow into the intracellular compartment. As a result, the electrical current preferentially travels through extracellular pathways, making the measurement more sensitive to changes in extracellular and interstitial fluid distribution as well as vascular blood volume. Consequently, the obtained impedance parameters primarily reflect alterations in extracellular tissue fluid dynamics rather than intracellular properties [33,34]. Two electrodes were positioned 2 cm paravertebrally on the right side at the level of the first lumbar vertebra (L1), and two additional electrodes were placed at the level of the fourth lumbar vertebra (L4). This configuration enabled assessment of electrical tissue properties in the TLF region. BIA values were interpreted as indicators of local tissue hydration and extracellular fluid distribution (Figure 1) [24].

In healthy individuals with no abnormal body shape, no fluid imbalance, and a BMI between 16 and 34 kg/m^2^, BIA provides reliable information on soft-tissue impedance [35]. The measurement error in standard whole-body BIA is typically less than ±1.2 Ω (±2%), with excellent test–retest reliability (ICC > 0.85) and a minimum detectable change (MDC) between 2.0 and 3.25 Ω [36]. In localized bioelectrical impedance analyses, even higher reliability values (ICC: 0.97–1.0) were observed under standardized measurement conditions, with inter-operator errors of <1–3% [37].

Subjective subacute/chronic stress over the past four weeks was assessed using the validated 10-item version of the PSS-10. Participants rated their experiences of stress over the preceding four weeks on a Likert scale ranging from 0 to 4. The total score ranged from 0 to 40, with higher scores indicating higher perceived stress levels [30,38].

Dynamic interactions between autonomic regulation and tissue properties were quantified using CRQA. This nonlinear time-series method evaluates the recurrence of similar states between two signals over time and allows for the detection of dynamic coupling. Cross-recurrence plots were generated for the HRV and BIA time series (which were downsampled to 1 Hz, corresponding to 300 data points over a 5-min period), and the parameters recurrence rate, determinism, entropy and mean line length were extracted [39]. The application of CRQA for time-series comparisons of biophysiological signals has been previously evaluated and described in detail in Brandl et al., 2026 [27]. In brief, CRQA was conducted as described in the original methodological framework of Wallot and Leonardi [39] to quantify the temporal coupling between the synchronized physiological signals. Phase-space reconstruction was performed using an embedding dimension of 1 and a delay of 1, while a recurrence radius of 0.2 defined the threshold for identifying recurrent states. Following common practice in CRQA, minimum diagonal and vertical line lengths were set to two points (minimum diagonal line = 2, minimum vertical line = 2) to exclude isolated recurrence points likely attributable to noise, and recurrences were evaluated across the entire time series without a Theiler window (tw = 0) and in both temporal directions (side = “both”). Artifacts were identified through visual inspection of the raw signals, and segments affected by noise, movement artifacts, or signal loss were excluded from further analysis.

To reduce the dimensionality of the data and summarize these parameters into a single metric, a principal component analysis (PCA) was performed, considering eigenvalues greater than 1 and using VARIMAX rotation. The resulting principal component (PCAC) was used as a composite measure of system coupling between HRV and BIA.

### 2.3. Statistical Analysis

Descriptive statistics were calculated and are reported as means, standard deviations (SD) and 95% confidence intervals (CI). Differences between the exam condition and the control condition were analyzed using paired *t*-tests for HRV, BIA, PSS-10 scores, and the PCAC.

Effect sizes were calculated using Cohen’s d and interpreted as “small” (d = 0.20–0.49), “medium” (d = 0.50–0.79), and “large” (d ≥ 0.80).

The effects of condition (PCAC control vs. exam), perceived stress (PSS-10 score), and their interaction were analyzed using a linear mixed-effects model. Participant-specific random intercepts were included to account for within-subject dependency of observations. The PSS-10 score, reflecting perceived stress during the previous month, was entered as a continuous covariate.

The assumptions for parametric tests were met for all hypothesis tests. Statistical significance was set at a level of *p* < 0.05. All statistical analyses were performed using Jamovi (Version 2.6) and R (Version 4.5.2).

## 3. Results

A total of 18 participants (nine female and nine male) were included in the analysis (age: 36.1 years, SD = 11.6, 95% CI: 30.26–41.83; BMI: 28.7, SD = 10.4, 95% CI: 22.53–33.90).

### 3.1. Cross-Recurrence Quantification Analysis

**Figure 2:**
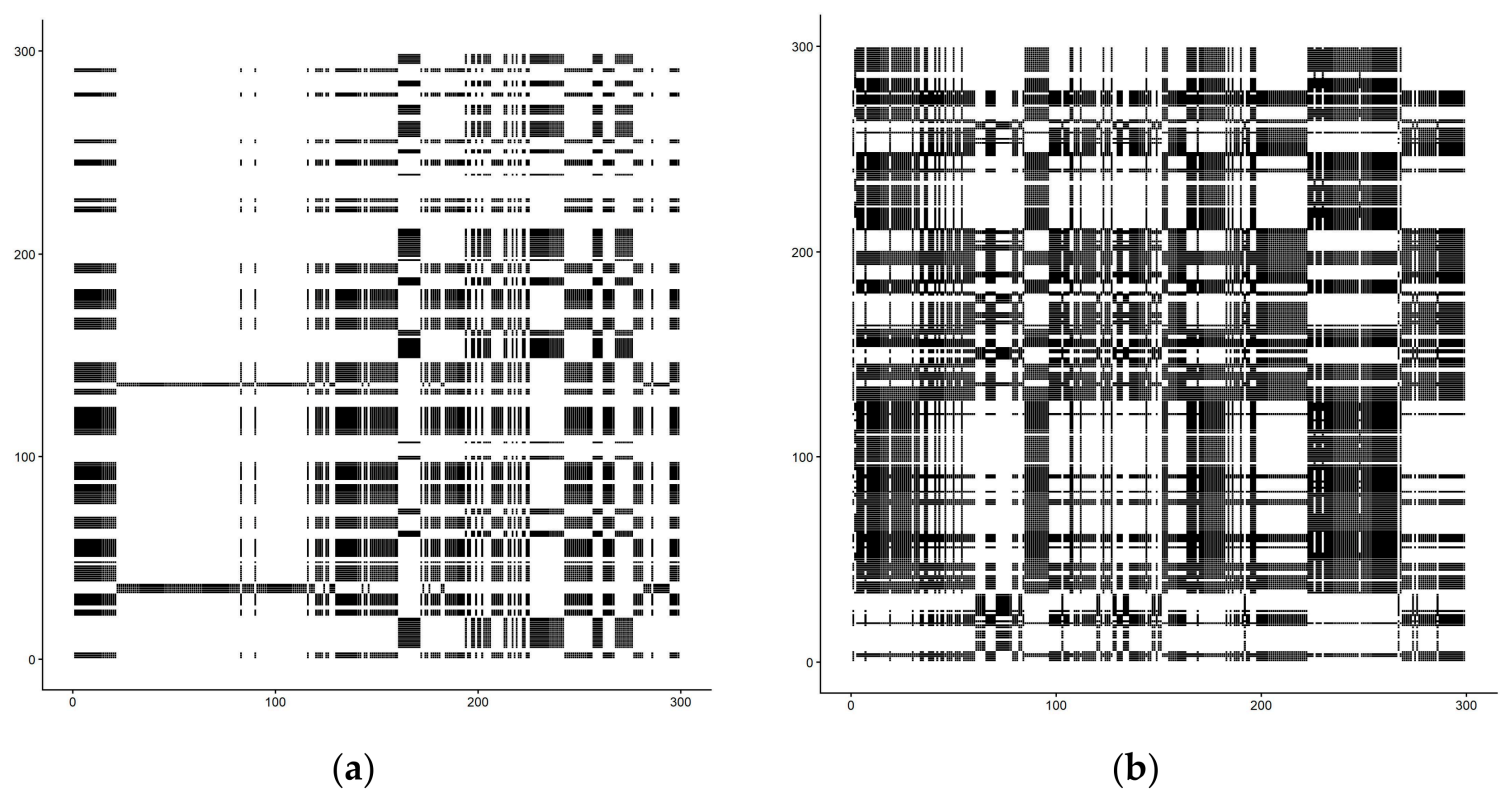
Cross-correlation plot of the exam conditions. (**a**) Cross-correlation plot of the control condition. (**b**) Cross-correlation plot of the exam condition. Black squares indicate quantifiable correlations between HRV and BIA. The plots show data from a representative participant.

The CRQA revealed clear differences in the dynamic interaction between HRV and BIA between the two conditions (Figure 2).

Visual inspection of the cross-recurrence plots indicated a higher density of recurrence points during the exam condition compared to the control condition, suggesting an increase in shared dynamic patterns between the two physiological signals. Visually, there was a clear increase in recurrences under the exam condition (Figure 2b) compared to the control condition (Figure 2a).

The CRQA parameters were reduced using PCA, and factor separation was maximized through a VARIMAX rotation, resulting in a component that explained 81.1% of the variance, namely PCAC, with high loadings on RR, DET, L, and ENTR (Table 1 and Table 2).

**Figure 3:**
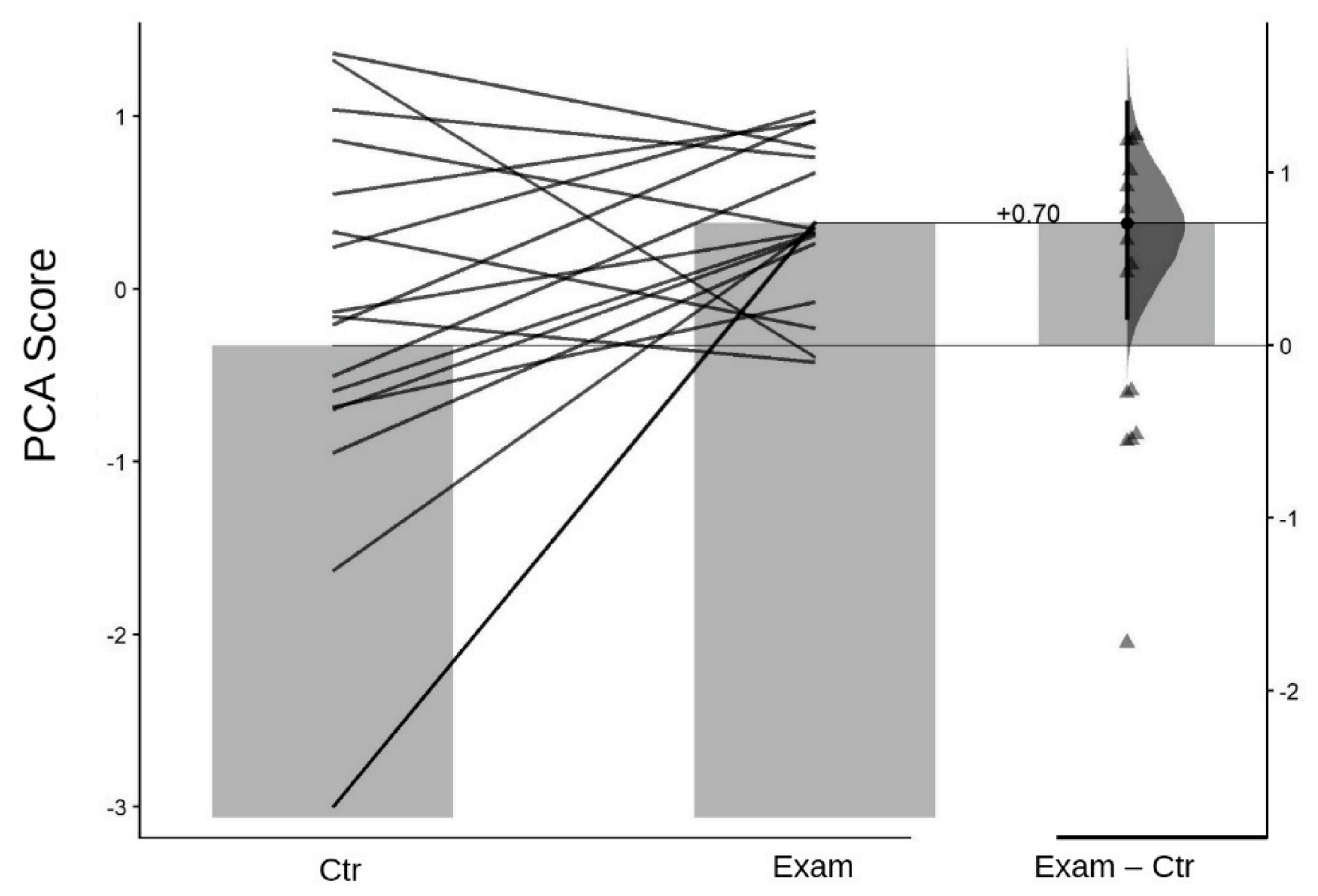
Estimation Plot of the PCA Component of the CRQA. The plot shows the pairwise comparisons between the control condition (Ctr) and the exam condition (Exam). Lines on the left side and triangles on the right side of the diagram represent the raw data points of a single participant. The right-hand side of the plot displays the results of the paired *t*-test, the mean difference (0.7), and its 95% confidence interval.

The PCA results presented here suggest that a large portion of the variability in the recurrence metrics can be summarized by a single factor describing the strength and consistency of the interaction between HRV and BIA. The mean value increased from −0.33 (SD = 1.27, 95% CI: −0.96–0.31) in the control condition to 0.38 (SD = 0.38, 95% CI: 0.16–0.60) in the exam condition. The paired *t*-test of the PCAC revealed significant differences between the conditions, t_17_ = −2.27; *p* = 0.037; Cohen’s d = −0.53. Figure 3 shows the estimation plot.

### 3.2. Heart Rate Variability

**Figure 4:**
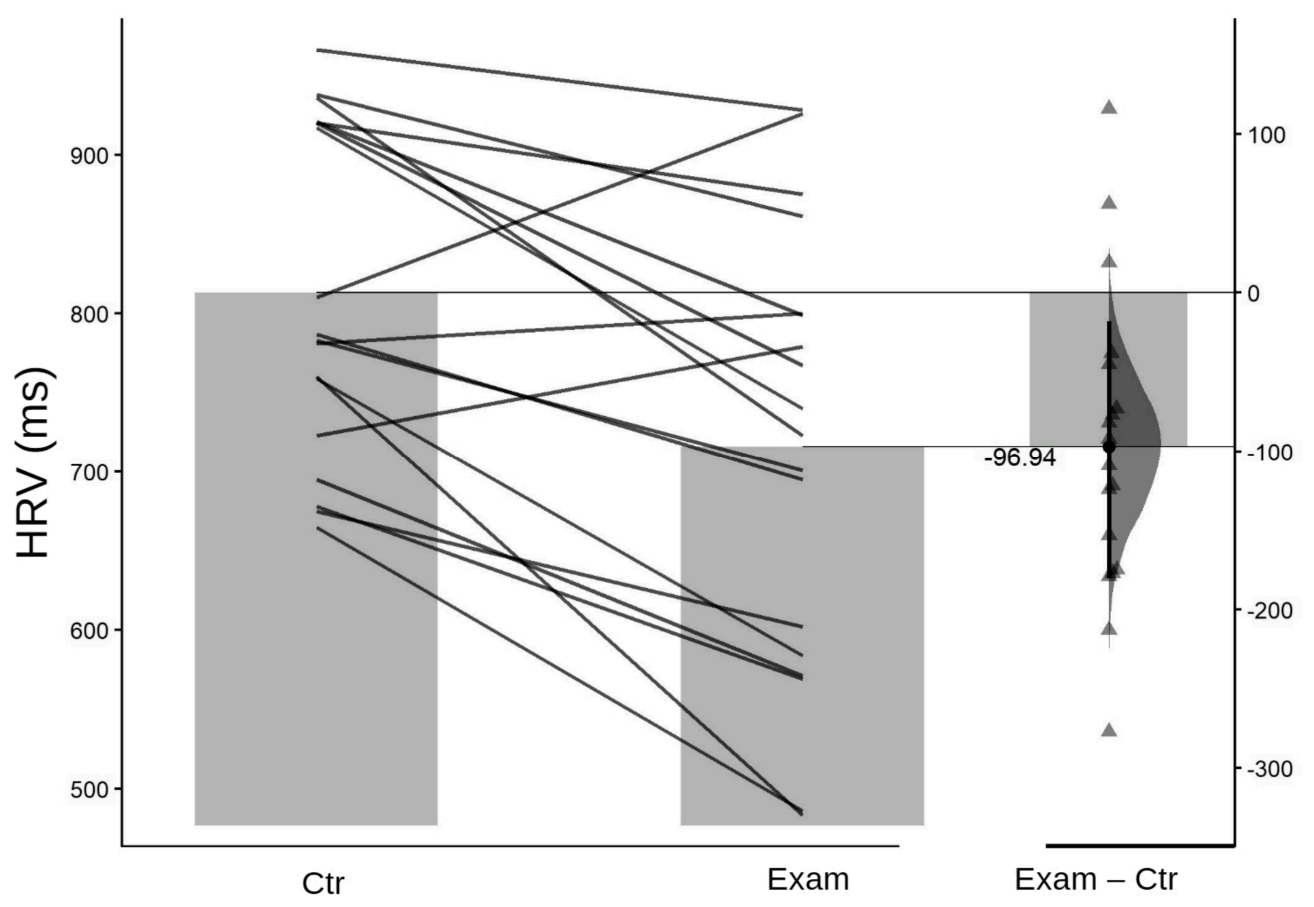
Estimation Plot of the HRV. The plot shows the pairwise comparisons between the control condition (Ctr) and the exam condition (Exam). Lines on the left side and triangles on the right side of the diagram represent the raw data points of a single participant. The right-hand side of the plot displays the results of the paired *t*-test, the mean difference (−96.94 ms), and its 95% confidence interval.

Mean RR intervals decreased from 813 ms (SD = 105 ms, 95% CI: 761–865) in the control condition to 716 ms (SD = 141 ms, 95% CI: 646–786) during the exam condition. The paired *t*-test for HRV revealed significant differences between the test and control conditions, t_17_ = 4.29; *p* < 0.001; Cohen’s d = 1.00. Figure 4 shows the estimation plot (Figure 4).

### 3.3. Bioelectrical Impedance Analysis

**Figure 5:**
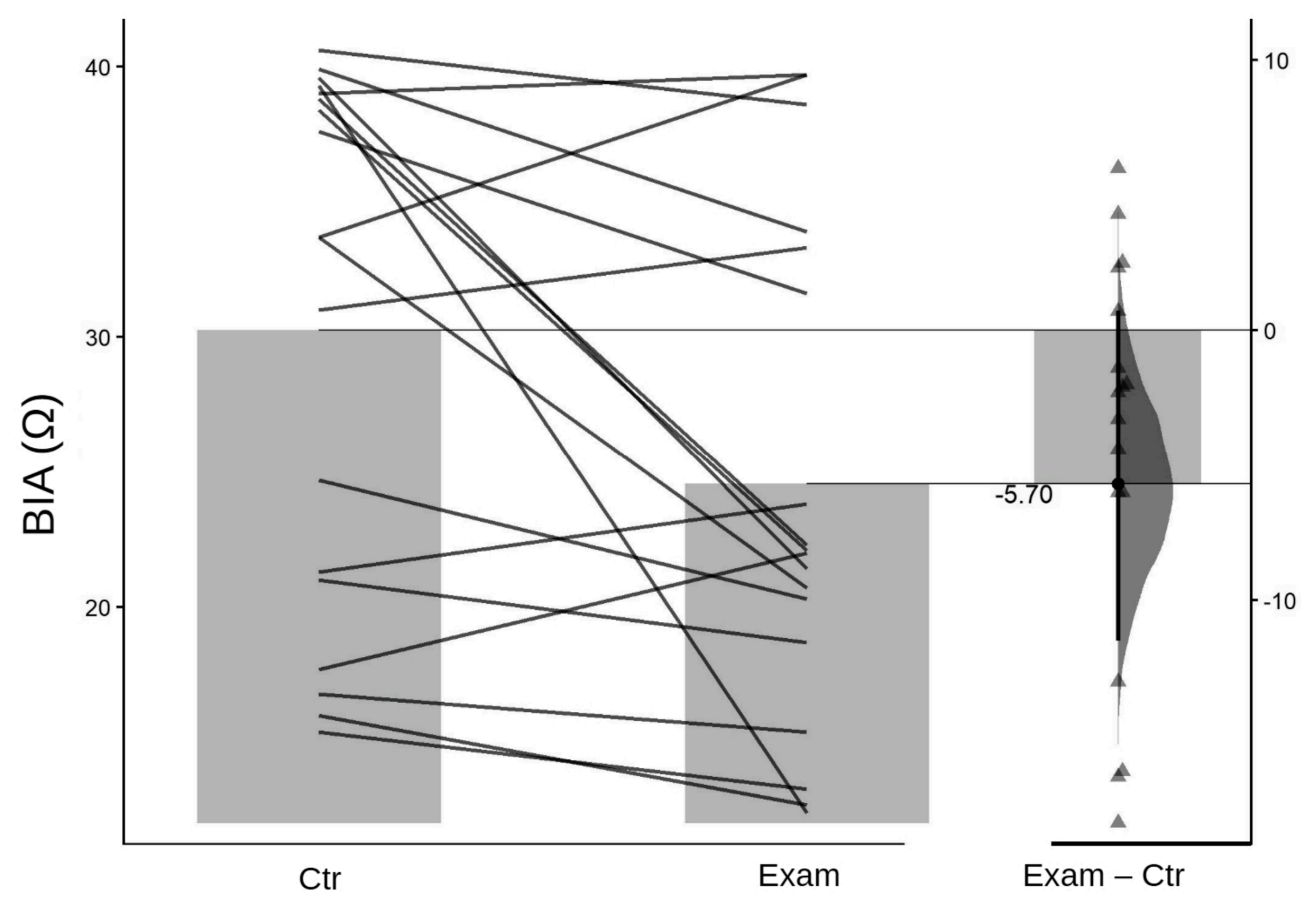
Estimation Plot of the BIA. The plot shows the pairwise comparisons between the control condition (Ctr) and the exam condition (Exam). Lines on the left side and triangles on the right side of the diagram represent the raw data points of a single participant. The right-hand side of the plot displays the results of the paired *t*-test, the mean difference (−5.7 Ω), and its 95% confidence interval.

The mean impedance was 30.25 Ω (SD = 9.77 Ω, 95% CI: 25.4–35.1) in the exam condition and decreased to 24.55 Ω (SD = 9.28 Ω, 95% CI: 19.9–29.2) during the exam condition. The paired *t*-test of the BIA revealed significant differences between the test and control conditions, t_17_ = 2.70; *p* = 0.015; Cohen’s d = 0.64. Figure 4 shows the estimation plot (Figure 5).

### 3.4. Perceived Stress

**Figure 6:**
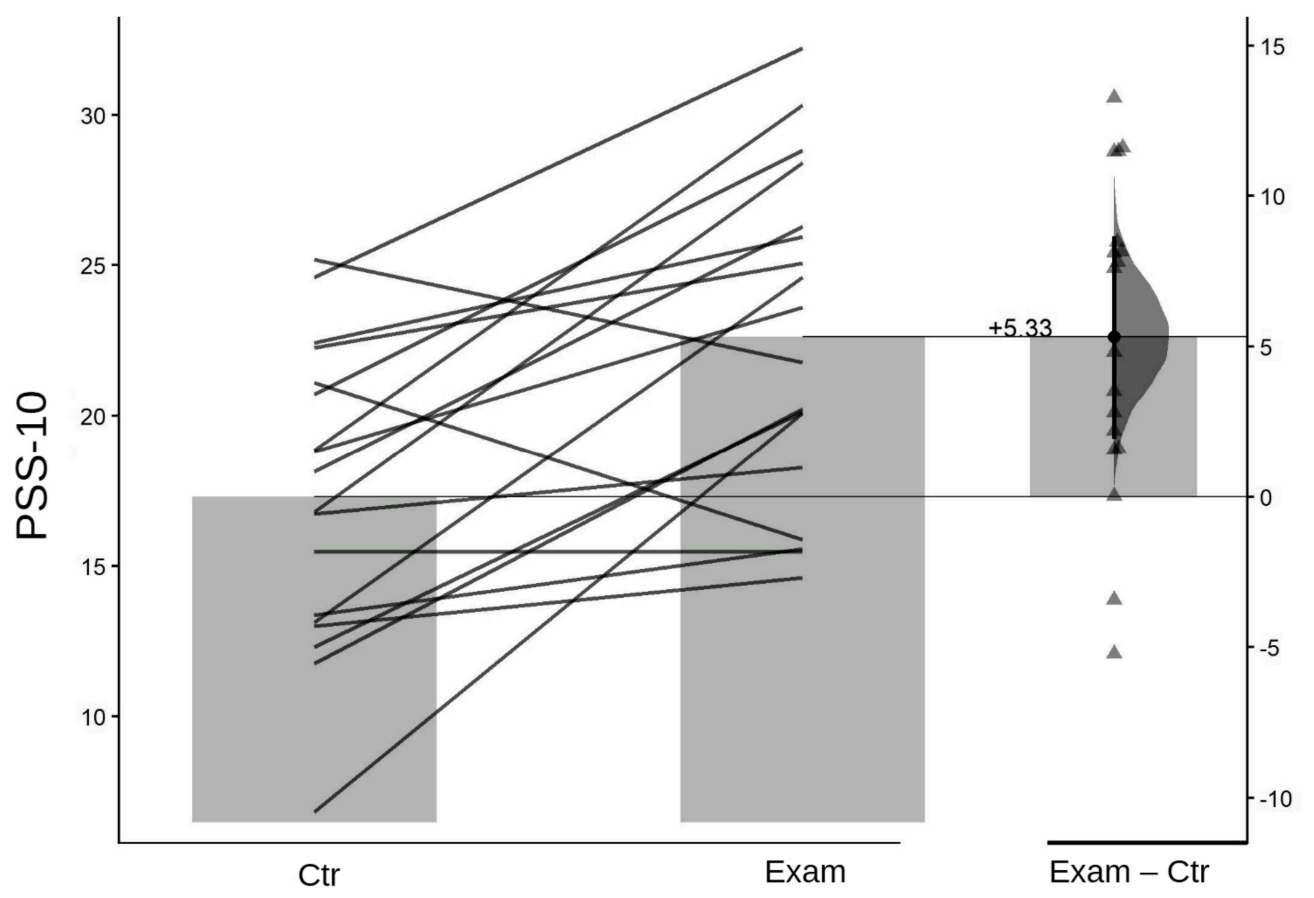
Estimation Plot of the PSS-10 scores. The plot shows the pairwise comparisons between the control condition (Ctr) and the exam condition (Exam). Lines on the left side and triangles on the right side of the diagram represent the raw data points of a single participant. The right-hand side of the plot displays the results of the paired *t*-test, the mean difference (5.33), and its 95% confidence interval.

Mean scores increased from 17.30 (SD = 4.96, 95% CI: 14.8–19.8) in the control condition to 22.63 (SD = 5.46, 95% CI: 19.9–25.3) during the exam condition. The paired *t*-test of the PSS-10 scores revealed significant differences between the test and control conditions, t_17_ = 4.26; *p* < 0.001; Cohen’s d = 1.00. Figure 5 shows the estimation plot (Figure 6).

### 3.5. Interaction of Condition and Perceived Stress

The model converged successfully and explained 25.8% of the variance through fixed effects (marginal R^2^ = 0.258) and 32.9% of the total variance when random effects were included (conditional R^2^ = 0.329). Table 3 shows the fixed main and interaction effects, and Table 4 shows the parameter estimates.

## 4. Discussion

To the authors’ knowledge, this study is the first to investigate the effects of acute psychosocial stress on autonomic regulation, tissue hydration of the lumbar spine, and their dynamic interaction. The findings demonstrate that a real-life examination setting induces significant physiological and subjective changes, including reduced HRV, decreased BIA, increased PSS-10 scores, and strengthened dynamic coupling between these systems as assessed by CRQA. These results support the hypothesis that acute stress induces coordinated, system-wide adaptations involving both central autonomic regulation and peripheral myofascial tissue dynamics.

### 4.1. Study outcomes

One of the central findings regarding our primary hypothesis of this study is the significant increase in recurrence measures derived from CRQA, indicating a stronger dynamic interaction between HRV and BIA under the exam condition. This pattern may reflect a heightened integration of physiological processes, which can be interpreted as an increase in what may be hypothetically termed “autonomic-fascial coupling.” From a physiological perspective, this finding is consistent with models proposing that stress-induced activation of the ANS modulates peripheral tissues via changes in microcirculation and extracellular matrix dynamics [7,9,17]. The TLF, due to its dense innervation and close relationship with autonomic pathways, is particularly susceptible to such regulatory influences [13,40,41]. As a result, acute stress may lead to synchronized fluctuations in autonomic signals and tissue properties, which are captured in the present study as increased recurrence patterns in the CRQA. The observed increase in recurrence patterns further indicates that the system exhibits more complex yet ordered dynamics during stress. This may reflect the emergence of coordinated regulatory processes aimed at maintaining homeostasis under challenging conditions. Similar findings have been reported in studies examining nonlinear physiological dynamics, where stress is associated with increased synchronization and structured variability across systems [39,42]. In this context, the present results could be interpreted as reflecting a dynamic interplay between autonomic regulation and fascial tissue properties under real-life stress conditions.

The significant reduction in HRV observed during the exam condition aligns with established findings in stress research. Acute psychosocial stressors, particularly those involving social evaluation and performance pressure, reliably induce sympathetic activation and vagal withdrawal [7,43]. This shift in autonomic balance is reflected in reduced variability in RR intervals, which is widely recognized as a marker of decreased regulatory flexibility [23]. The large effect size observed in the present study highlights the robustness of the response to the exam condition within the experimental paradigm. In addition, the increased variability in HRV across individuals suggests heterogeneity in autonomic stress reactivity, which has been reported in previous literature [44,45]. These interindividual differences may reflect variations in stress appraisal, coping strategies or baseline autonomic tone, and point to the importance of considering individual variability in stress-related research. Importantly, when interpreted in combination with the CRQA findings, the reduction in HRV not only reflects a change in autonomic balance but also appears to be functionally linked to peripheral tissue dynamics. This supports an integrative model in which central autonomic changes are directly associated with alterations in tissue physiology via neurovascular mechanisms.

The significant decrease in BIA impedance during the exam condition suggests that acute stress is associated with changes in the electrical properties of the lumbar tissue, which can be interpreted as alterations in local hydration and fluid distribution. Given that electrical conductivity in biological tissues is strongly influenced by water content and electrolyte distribution, lower impedance values are typically indicative of increased fluid availability [24]. In the context of fascial physiology, such changes may reflect modifications in the extracellular matrix, particularly in the hyaluronan-rich interstitial space that contributes to lubrication and tissue glide [19,46]. Acute stress-induced sympathetic activation may influence microvascular tone and capillary filtration, thereby altering interstitial fluid balance within the fascia [17,47]. These changes may have immediate consequences for the viscoelastic properties of the tissue and its mechanical function. Experimental studies have demonstrated that alterations in hydration could modify fascial sliding properties and influence mechanical behavior, including stiffness and shear capacity [13,48]. The present findings support the hypothesis that stress-related fluid shifts occur not only over longer time scales but can also manifest acutely during real-life stress exposure. This adds to the growing body of evidence suggesting that the myofascial system is highly dynamic and sensitive to neurophysiological regulation.

The PSS-10 results in the present study provide a meaningful classification of subjective subacute/chronic stress over the past four weeks within the context of existing literature on student populations. The mean score of 17 in the control condition falls within the range typically reported during regular academic periods and can therefore be interpreted as reflecting a moderate and expected level of stress [38,49]. This supports the validity of the control condition as a realistic representation of everyday academic demands. In contrast, the increase to a mean PSS-10 score of 23 during the exam condition indicates a substantial elevation in perceived stress. This value is consistent with findings from prior studies showing that examinations represent one of the most potent stressors in higher education, with reported scores commonly ranging between 22 and 26 points [50,51]. The observed difference of approximately six points therefore represents a meaningful and practically relevant increase in subjective stress, confirming that the applied stress paradigm elicited a robust and clinically significant stress response. This provides an important context for interpreting the accompanying physiological changes within a biopsychosocial framework.

Although not statistically significant (*p* = 0.073), the magnitude and direction of the estimated effect of the Interaction of Condition and Perceived Stress—our secondary hypothesis—suggest that perceived stress may influence the relationship under investigation. This means that the individual level of perceived subacute/chronic stress could influence the dynamics of a hypothetical autonomic-fascial coupling due to different stressors. Chronic stress is associated with sustained activation of the sympathetic nervous system and the HPA axis, leading to prolonged exposure to cortisol and catecholamines. Over time, these adaptations may contribute to downregulation of adrenergic receptors, reduced sympathetic responsiveness, and impaired function of the stress system [52,53,54]. In particular, it is believed that stress necessitates a reorganization of system network couplings, which may apply here [55]. Follow-up studies are therefore required to further investigate the trend observed here.

### 4.2. Implications for Practice

The findings of this study have important implications for clinical physical therapy, particularly within a biopsychosocial framework. The assumed autonomic-fascial coupling suggests that psychosocial stressors may influence not only central regulation but also peripheral tissue properties in a rapid and measurable way. This supports the notion that interventions targeting autonomic regulation, such as breathing techniques, relaxation strategies or movement-based therapies, may have downstream effects on tissue hydration and mechanical function.

Given the established role of the TLF in force transmission and its significance in low back pain [12,56], stress-induced changes in tissue properties may contribute to pain susceptibility or functional impairment. Integrating psychological stress regulation strategies into physical therapy could therefore enhance treatment outcomes, particularly in populations with chronic musculoskeletal conditions or stress-related disorders.

### 4.3. Limitations

A primary limitation of the present study concerns the relatively small and heterogeneous sample size. With only 18 participants and a wide range of anthropometric characteristics (age, BMI) the generalizability of the findings is limited. Interindividual variability in physiological responses, particularly in HRV and BIA measures, may therefore have influenced the results. However, the repeated-measures design partially mitigates this limitation by allowing each participant to serve as their own control, thereby reducing between-subject variability. Future studies should include larger and more homogeneous samples to improve external validity and enable subgroup analyses based on relevant factors such as age, fitness level or stress reactivity.

A second limitation relates to the potential influence of uncontrolled confounding variables, particularly in HRV measurements. Factors such as respiration rate, hydration status, sleep quality, caffeine intake and circadian rhythms are known to affect autonomic regulation and may have influenced the observed results. In the present study, these variables were not strictly controlled in order to preserve ecological validity and to reflect real-life conditions of examination stress. While this approach strengthens the external validity of the findings, it may have introduced additional variability. Future research could address this limitation by combining ecologically valid designs with partial standardization of key confounding variables or by statistically controlling for them.

Third, the exam and control conditions differed in several respects beyond stress level. Because all participants completed the exam before the control condition, effects of stress cannot be fully separated from potential order or habituation effects. Moreover, speech and muscle activity may have differed under exam and control conditions, so the observed physiological changes should be interpreted as reactions to the overall context rather than as evidence of a specific causal effect of psychological stress alone. Future studies should take this into account and employ counterbalanced designs.

The BIA in the lumbar region also presents methodological limitations. Stress-induced sweating may alter skin conductance and thus affect impedance readings. Although this effect is likely less pronounced in the lower back compared to areas with higher sweat gland density [57,58], it cannot be completely excluded. In the present study, this limitation was minimized by the choice of measurement location and by using a measurement frequency (12 kHz) at which superficial skin hydration effects are reduced. Nevertheless, future studies could improve measurement accuracy by using ultrasound-guided needle electrodes directly in the region of interest.

Another limitation concerns the methodological characteristics of CRQA. Although it is a powerful tool for analyzing nonlinear interactions between physiological systems, it is sensitive to parameter selection, including embedding dimension, delay and threshold settings. While the present study applied standardized procedures and reduced dimensionality using PCA, it remains unclear to what extent the results are robust to variations in these parameters. Moreover, CRQA does not allow for causal inference, but only captures the strength and structure of dynamic coupling. This limitation was addressed by interpreting CRQA results within a broader physiological framework; however, future research should explore the stability of findings across parameter settings and combine CRQA with causal modeling approaches.

A further limitation is the use of only two measurement time points, corresponding to the exam and control conditions. This design captures condition-specific differences but does not allow for the analysis of temporal dynamics of the stress response, such as anticipatory activation, peak response or recovery processes. Future studies should incorporate longitudinal or repeated time-series designs to better characterize the temporal trajectory of stress-related autonomic and tissue responses. Furthermore, future studies should incorporate measures of acute psychological states, such as exam-related anxiety, in addition to evaluating the subacute and chronic stress of interest with the PSS-10 (e.g., using the Test Anxiety Inventory, TAI).

## 5. Conclusions

In summary, the present study suggests that acute exam stress is associated with coordinated changes in autonomic regulation and lumbar tissue properties, probably accompanied by increased coupling of HRV and BIA, which has been hypothesized as autonomic-fascial coupling. These findings are consistent with a biopsychosocial model in which stress-related processes influence both central and peripheral physiological mechanisms. Further research is needed to clarify these interactions and their potential relevance for clinical physical therapy.

## Figures and Tables

**Table 1:** Component Loadings.

	Component	Uniqueness
RR	0.843	0.2888
DET	0.898	0.1943
L	0.886	0.2145
ENTR	0.971	0.0567

**Table 2:** Component eigenvalue.

Component	Eigenvalue	% of Variance	Cumulative %
1	3.2456	81.14	81.1
2	0.3886	9.71	90.9
3	0.2912	7.28	98.1
4	0.0745	1.86	100.0

**Table 3:** Fixed Effects Omnibus Tests.

	F	df, df (Res)	*p*
condition	9.71	1, 22.4	0.005
PSS-10	3.73	1, 27.7	0.064
condition × PSS-10	3.52	1, 24.6	0.073

**Table 4:** Parameter estimates.

				95% CI				
Names	Effect	Estimate	SE	Lower	Upper	df	t	*p*
(Intercept)	(Intercept)	−0.12	0.18	−0.471	0.239	21.8	−0.67	0.511
time1	t1 − t0	1.01	0.33	0.349	1.679	22.4	3.12	0.005
PSS-10	PSS-10	−0.06	0.03	−0.120	0.003	27.7	−1.93	0.064
time1 × PSS-10	(t1 − t0) × PSS-10	0.11	0.06	−0.010	0.225	24.6	1.88	0.073

## Data Availability

The raw data supporting the conclusions of this article will be made available by the authors on request.

## Abbreviations

The following abbreviations are used in this manuscript:

ANS	Autonomous nervous system
BIA	Bioelectrical impedance analysis
BMI	Body-Mass-Index
CRQA	Cross-recurrence quantification analysis
HPA	Hypothalamic-pituitary-adrenal
HRV	Heart rate variability
PCA	Principle component analysis
PCAC	Resulting component of PCA
PSS-10	Perceived-Stress-Scale
TLF	Thoracolumbar fascia
